# How is global change affecting Chagas disease landscapes?

**DOI:** 10.1590/0074-02760210479chgsa

**Published:** 2022-05-27

**Authors:** Ricardo E Gürtler

**Affiliations:** 1 Universidad de Buenos Aires, IEGEBA-CONICET

Chagas disease (CD), a neglected tropical disease (NTD) intimately linked to social vulnerability, has been targeted for elimination as a public health problem in the 2021-2030 road map set by the World Health Organization (WHO).^1^ This implies the interruption of transmission through four routes (vectorial, transfusional, congenital and transplant) and 75% antiparasitic treatment coverage of eligible cases. OPS/WHO^2^ recently set specific requirements for such goal, including the interruption of transmission of human infection with *Trypanosoma cruzi*, virtual suppression of all intradomiciliary colonies of the target vector species, and other aims according to the vectors’ main biological features. The 2021-2030 road map reports considerable progress since the last review, and calls for accelerated programmatic action. As its 2007 and 2012 predecessors, the new report “failed to emphasise several key challenges that are currently undermining these achievements and that must be urgently addressed in order to move to the next stage: ensuring the long-term and sustainable control of this devastating disease”.^3^

The broad-scope, timely review conducted by Rita CM de Souza and expert colleagues fill this gap by identifying key challenges to CD elimination and linking the disease to the 2030 Agenda for Sustainable Development. Of the 17 sustainable development goals (SDG), they identify 16 to which CD prevention would contribute to and benefit from in a two-way road. For example, promoting healthy housing and quality education will minimise vector-borne transmission and improve health. Better road infrastructure will increase opportunities for trade and access to health-care services in remote rural areas. Similarly, resolution of social conflict and violence would cease to interfere the deployment of disease control interventions in rural, peri-urban and urban settings across the continent.

The review treads carefully over the multiple biological, environmental and socio-economic drivers of CD vector control through Latin America. A large number of *T. cruzi*-infected triatomine species invade human habitations and feed on humans and domestic animals. Perhaps large-scale deforestation and concomitant reductions in host abundance displaced some species from their natural habitats, while some may be in the process of adapting to more stable habitats with a better host supply (domestication). For most of the triatomine species reviewed, there is scant information on abundance, habitat selection, blood-feeding patterns (including contact rates with humans), infection rates, vectorial competence, parasite genotypes, and response to insecticide spraying or other control tools. Do they cause human infection with *T. cruzi*, and if so, how often, where and when? What is their relative share of human infection and disease?

The environmental drivers of CD interact dynamically. Few studies addressed the effects of deforestation and vertebrate diversity (“dilution effect”) on vector abundance and infection at relevant spatial scales, and little is known on their effects on parasite transmission rates and human infection with *T. cruzi*. The issue of intrusive triatomine species not vulnerable to classic insecticide spraying operations, such as *Rhodnius pallescens*, and of many other triatomines with widespread sylvatic foci that colonise human habitations, such as *Triatoma brasiliensis*, still require innovative approaches. The process of (peri)urbanisation of triatomine species that invade and sometimes colonise the peripheral slums of small towns and big cities is receiving increasing attention from researchers. While this process is genuinely relevant from an evolutionary perspective, we need to translate these house invasion events into human infection risks and assess the need of preventative measures. The environmental drivers underlying the unexpected emergence of pyrethroid resistance in *Triatoma infestans* in the late 1990s are still a matter of speculation and some concern beyond Argentina and Bolivia. The available options for replacing pyrethroids (e.g., organophospate insecticides) have become increasingly questioned or unacceptable by the parties involved.

Global change is the larger phenomenon that includes climate, environmental and social change. The growing demand for resources of an increasing human population go hand in hand with land-use change, steady rural-to-urban migration, and climate change. The review singles out two expressive study cases. The African oil palm-*Rhodnius prolixus* matrimony in Colombia and the soybean-displacement in central Argentina illustrate two opposite effects of rural development based on intensified agriculture. The former created a much larger potential threat where there was a minor one, while the soybean expansion to formerly semiarid areas displaced the campesino populations to urban peripheries and wiped out the traditional mud-and-thatch houses frequently infested with *T. infestans*. Our long-term intervention programme in Pampa del Indio, in the Argentine Chaco, has recorded similar trends in displacement and land-use change. Has relocation increased the access of CD patients to adequate health-care attention, employment and healthy housing? Here, the intersections among rural development, housing quality, employment, migration, and disease status provide fertile grounds for research and action.

Social change encompasses changes in human interactions and institutional arrangements. The review provides some examples of declining or extinct CD control programmes in areas with evidence of disease transmission. I believe the list would be much longer if the basic information were open to public scrutiny. In line with the current WHO road map for the elimination of CD as a public health problem, a sound step would be to inventory the current status and track records of triatomine control programmes in the affected countries. Tonn (1980) made such appraisal on behalf of WHO;^4^ it provided a valuable reference for comparative studies that seek to understand the current epidemiological status of CD. Likewise, the history of the Southern Cone Initiative for CD control produced by Antonio Carlos Silveira and colleagues^5^ is a data-rich source of the undertakings and achievements of vector control programmes in this region.

Will climate change affect the distribution and frequency of human CD? The net outcome of a complex web of interactions is uncertain, more so because there is little guidance on how to assign relative weights to each process (e.g., deforestation, biodiversity loss, temperature increase), and even the sign of change may be reversed by other processes. While there is consensus that the expected increase in temperature will modify the current distribution and fitness of triatomine species and their sylvatic hosts, the pathways to parasite transmission and human infection are affected by first-order social processes. For example, the Mennonite colonies that settled in the arid Paraguayan Central Chaco over 1920-1940 heavily invested in rural development and progressively altered the disease landscape in fundamental ways. The local levels of house infestation with *T. infestans* some decades ago were much lower levels than elsewhere in underdeveloped rural areas of the Argentine and Bolivian Chaco. In Pampa del Indio, the odds of human infection increased with increasing household social vulnerability and infected-bug abundance, but the magnitude of these effects was modified by household mobility.^6^

The (re)emergence of infectious diseases over the last 40 years is a strong component of global change, with both direct and indirect effects on the current and future status of CD. The Americas have experienced recurrent dengue virus outbreaks since the 1980s, followed by the recent emergence of Zika and Chikungunya virus, all transmitted by *Aedes aegypti*. This new front, running in parallel to the decentralisation of health-care services, diverted the attention and budget allocations of vector control programmes to the more demanding, politically relevant urban populations under epidemic outbreaks with a deadly toll.

The Coronavirus disease 19 (COVID-19) pandemic is the new global disruptor, causing immediate effects on the biota and human welfare, and with vast socio-economic sequelae lying ahead. For one specific example, triatomine control operations in endemic rural areas of the Argentine Chaco were downsised or worse since mid-March 2020 and the prospects for 2022 are gloomy. Consequently, partial recovery of *T. infestans* populations held in check by government-sponsored insecticide spray teams is the most likely event. Rather than the notification aspect of vector surveillance (which can be maintained through cell phones), the response components mediated by health-care personnel are the most affected ones, including diagnosis and treatment. The call for strengthening vector control programmes^7^ and implementing appropriate measures adapted to the new scenario is more in point than ever before.

Comments on the article: Souza RCM, Gorla DE, Chame M, Jaramillo N, Monroy C, Diotaiuti L. Chagas disease in the context of the 2030 agenda: global warming and vectors. Mem Inst Oswaldo Cruz. 2022; 117: e200479.
